# Interfacial Charge Transfer for Enhancing Nonlinear Saturable Absorption in WS_2_/graphene Heterostructure

**DOI:** 10.1002/advs.202306096

**Published:** 2024-01-15

**Authors:** Yiduo Wang, Yingwei Wang, Changyong Lan, Li Zhou, Jianlong Kang, Wanxin Zheng, Tianyu Xue, Yejun Li, Xiaoming Yuan, Si Xiao, Heping Li, Jun He

**Affiliations:** ^1^ Hunan Key Laboratory of Nanophotonics and Devices School of Physics and Electronics Central South University Changsha 410083 China; ^2^ Hunan Key Laboratory for Super‐microstructure and Ultrafast Process School of Physics and Electronics Central South University Changsha 410083 China; ^3^ State Key Laboratory of Electronic Thin Films and Integrated Devices School of Optoelectronic Science and Engineering University of Electronic Science and Technology of China Chengdu 610054 China; ^4^ Center for High‐Pressure Science State Key Lab of Metastable Materials Science and Technology Yanshan University Qinhuangdao 066004 China

**Keywords:** 2D materials heterostructures, enhancement of saturable absorptions, Interfacial charge transfers, nonlinear optical responses

## Abstract

Interlayer charge‐transfer (CT) in 2D atomically thin vertical stacks heterostructures offers an unparalleled new approach to regulation of device performance in optoelectronic and photonics applications. Despite the fact that the saturable absorption (SA) in 2D heterostructures involves highly efficient optical modulation in the space and time domain, the lack of explicit SA regulation mechanism at the nanoscale prevents this feature from realizing nanophotonic modulation. Here, the enhancement of SA response via CT in WS_2_/graphene vertical heterostructure is proposed and the related mechanism is demonstrated through simulations and experiments. Leveraging this mechanism, CT‐induced SA enhancement can be expanded to a wide range of nonlinear optical modulation applications for 2D materials. The results suggest that CT between 2D heterostructures enables efficient nonlinear optical response regulation.

## Graphical abstract

Saturable absorption modulation of 2D heterostructures is an important issue in the field of nonlinear optics of 2D materials. In this work, the mechanism of interfacial carrier transfer modulation on saturable absorption response by experimental and simulation methods are designed and verified, which provides guidance for the application of 2D materials in all‐optical and optoelectronic devices.

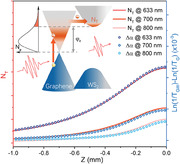

## 1 Introduction

By regulating light time‐domain features in laser resonators, saturable absorbers with large absorption range, recovery time, and modulation depth^[^ ^1^ ^]^ have become an indispensable tool for ultrashort pulse generation and other nonlinear photonics elements.^[^ ^2^ ^]^ Recently, 2D materials have been recognized as promising candidates for saturable absorption (SA) benefiting from their excellent SA properties, broad optical response, and ultrafast recovery time.^[^ ^1^, ^3^ ^]^ Monolayer graphene (Gr) exhibits broadband and ultrafast SA response.^[^ ^4^ ^]^ Nevertheless, the weak absorption (<3%) of graphene^[^ ^5^ ^]^ limits its modulation depth as SA. The direct bandgap character of monolayer transition metal dichalcogenides (TMDs) enables their intense absorption near its band edge.^[^ ^6^ ^]^ At the same time, it limits their broadband saturable absorption response and fast carrier recovery, thus hindering their SA performance to generate ultrashort pulses.

Constructing TMDs/Gr 2D heterostructure may address this challenge by combing the advantages of both and triggering the interfacial charge transfer (CT) effect. The photon‐generated carriers in WS_2_ can transfer to Gr with high efficiency,^[^ ^7^ ^]^ accelerating the carrier recovery speed of heterostructure. In addition, the intense carrier‐carrier scattering in Gr leads to the rapid (<100 fs) creation of thermalized Fermi‐Dirac (FD) distribution.^[^ ^8^ ^]^ The thermalized hot carrier in Gr has a high‐energy band tail. This enables the excited carrier to cross the barrier with higher energy and transfer to TMDs, which is so called photo‐thermionic emission (PTE) process.^[^ ^9^ ^]^ Chen et al^[^ ^10^ ^]^ proved that the quasi‐thermalized carriers can be effectively collected by WS_2_ after ultrafast intraband scattering (≈10 fs). Hence, the CT process in the Gr/TMDs interface is highly efficient regardless of the excitation photon energy, making the Gr/TMDs heterostructure an excellent optoelectronic platform.^[^ ^6^, ^11^ ^]^ Moreover, the interfacial defect states of Gr/TMDs heterostructures are considered to play a crucial role in the long‐lived charge separation,^[^ ^12^ ^]^ which can be fully controlled by gate experiments, demonstrating the potential of Gr/TMDs for electro‐optic modulation applications.^[^ ^13^ ^]^ To date, a large number of works have reported the excellent nonlinear optical response of the 2D heterostructure systems. On the one hand, collective nonlinear optical effects in hybrid 2D heterojunctions are difficult to exclude.^[^ ^3^, ^14^ ^]^ On the other hand, the mechanism of enhanced nonlinear optical response in vertical heterojunctions remains a tremendous challenge.^[^ ^15^ ^]^ That is, it remains unclear how the CT process affects the saturation absorption response.

**Figure 1:**
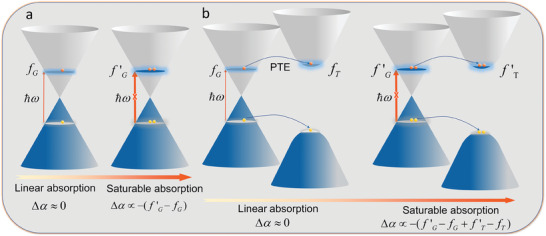
The photo‐thermionic emission (PTE) process induced the enhancement of saturable absorption in Gr/TMDs heterostructure. a) Pauli blocking induced saturable absorption in Gr, and the modulation depth Δα is negatively correlated with the difference between the distribution function at weak excitation (*f_G_*) and strong excitation (*f*′_*G*_).^[^ ^16^ ^]^ b) Saturable absorption in Gr/TMDs heterostructure with the excitation under the A exciton of TMDs, the enhanced modulation depth originated from the difference of distribution function in TMDs (*f*′_*T*_ − *f_T_*), which is due to the PTE process induced carrier transfer from Gr to TMDs.

The proposed concept of interfacial CT for enhancing nonlinear SA in atomically thin vertical stacks heterostructures is illustrated in **Figure** **1**. Due to the Pauli exclusion principle, the sample will produce a saturation absorption process at a higher light intensity, that is, its absorption coefficient decreases with an increase in light intensity, where the change of absorption coefficient of the sample (Δα, modulation depth) is negatively correlated with the change in hot carrier distribution.^[^ ^16^ ^]^ As shown in Figure 1a, the SA in Gr occurs when the excitation light fluence exceeds the linear absorption threshold value,^[^ ^3^, ^17^ ^]^ in which the absorption coefficient is negatively correlated with the distribution function in valence and conduction bands.^[^ ^16^ ^]^ In this case, the modulation depth of SA in Gr could be expressed as Δα∝ − (*f*′_*G*_ − *f_G_*). Here *f* represents the electron/hole transient distribution function according to the previous definition.^[^ ^16^ ^]^ $f^{\prime }$ and *f* correspond to the distribution functions with and without nonlinear optical response, respectively. In Figure 1b, when photocarrier generation arises in Gr from interlayer CT by PTE pathways,^[^ ^9^, ^10^ ^]^ the carrier distribution in WS_2_ can exhibit expected nonlinear optical response modulation. As a result, the difference in the distribution function of TMDs (*f*′_*T*_ − *f_T_*) can increase the modulation depth of Gr/TMDs heterostructure. Therefore, the modulation depth of SA in Gr/TMDs could be expressed as Δα∝ − (*f*′_*G*_ − *f_G_* + *f*′_*T*_ − *f_T_*), resulting in an enhanced modulation depth compared to the pristine one.

This work proposed and experimentally demonstrated that interfacial CT can enhance SA in Gr/TMDs heterostructure. The open aperture (OA) Z‐scan was employed to measure the nonlinear absorption of a well‐studied WS_2_/Gr system undergoing CT and compare it with that of pristine WS_2_ and Gr to quantify the enhancement. This result was further verified under different conditions, corresponding to sub‐bandgap photon excitation (*hν* < 2 eV, sub‐A‐exciton transition of WS_2_) and above‐bandgap photon excitation (*hν* > 2 eV, above‐A‐exciton transition of WS_2_). The experiments were corroborated with a calculation model based on PTE transfer and ab initio nonadiabatic molecular dynamics (NAMD) simulations. This study elucidates and validates the logical link between CT and nonlinear optical enhancement in Gr/TMDs heterostructure, which can be extended to other 2D van der Waals heterostructures, thus opening new avenues for nonlinear optical regulation strategy.

## 2 Results and Discussion

**Figure 2:**
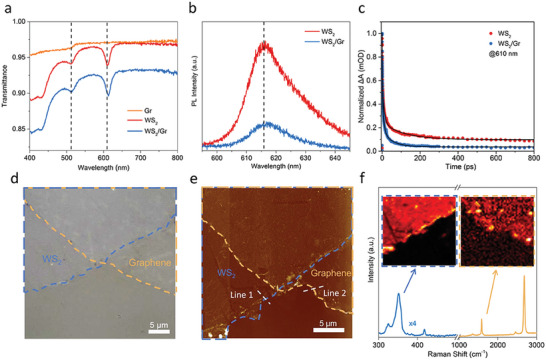
Characterization of WS_2_/Gr heterostructure. a) Transmission spectrum of WS_2_ monolayers, Gr, and WS_2_/Gr heterostructure. b) Photoluminescence (PL) spectrum of WS_2_ monolayers and WS_2_/Gr heterostructure. c) Normalized transient dynamics of the WS_2_/Gr heterostructure and WS_2_ with 400 nm pump and 610 nm probe, solid lines are the fitting curves with exponential decay function. d) Photograph and e) atomic force microscopy (AFM) topographic image of the WS_2_/Gr heterostructure on the sapphire substrate. The height profile along the dashed white line is shown in Figure S4 (Supporting Information). f) Raman spectra for pristine WS_2_ (blue), and Gr (orange). The inset image is the Raman mapping at 351 cm^−1^ and 1584 cm^−1^, which corresponds to the Raman fingerprint of WS_2_ and Gr.

The fabrication details of WS_2_/Gr heterostructure can be found in Supporting Information. Briefly, chemical vapor‐deposited (CVD) monolayer WS_2_ was transferred onto CVD Gr, which was grown on a transparency sapphire substrate (Figure S1a, Supporting Information). UVvis transmission spectroscopy (**Figure** **2**a) and photoluminescence (PL)spectroscopy (Figure 2b) were employed to characterize the static optical properties. Two intense exciton peaks at 610 nm (2.03 eV, A exciton) and 512 nm (2.42 eV, B exciton) of pristine WS_2_ were observed, corresponding to the two characteristic excitons of monolayer WS_2_.^[^ ^18^ ^]^ The pristine Gr exhibited a typical uniform and weakened absorption (≈3%) in the wide wavelength^[^ ^12^ ^]^ compared with pristine WS_2_ The A exciton peak of WS_2_/Gr heterostructure exhibited a redshift with certain broadening, which was due to the dielectric screening of Gr on WS_2_ and the CT from WS_2_ to Gr. This feature was also confirmed by PL quenching and redshift of WS_2_/Gr heterostructure (Figure 2b).

The ultrafast CT process in WS_2_/Gr heterostructure was further analyzed by transient absorption (TA) spectrum technique with a pump wavelength of 400 nm. WS_2_ and WS_2_/Gr showed similar initial TA signals at broadband probe wavelengths (Figure S2, Supporting Information). The TA signals were extracted at different wavelengths at 400 fs. It was found that under the same experimental conditions, the signal of WS_2_/Gr was only slightly larger than that of WS_2_. This indicated that the signal above the A exciton was mainly dominated by WS_2_, and the Gr signal only accounted for a small part of it, which was also confirmed by the separate TA experiment of Gr (Figure S3, Supporting Information). In addition, compared with the TA peak of WS_2_ at 610 nm, the TA peak of WS_2_/Gr was red‐shifted to 611 nm. Similar to the linear absorption spectrum (Figure 2a), this redshift was also due to the dielectric screening effect of Gr on WS_2_. On the other hand, the dynamic curves extracted at 610 nm showed obvious differences (Figure 2c). The dynamic curves were fitted with a three‐exponential decay form. As shown in Table S1 (Supporting Information), the first two lifetime values were very close, and they were assigned to the exciton formation process (≈1 ps) and the exciton‐exciton annihilation process (≈10 ps)^[^ ^19^ ^]^ respectively. However, the third longer lifetime shows a significant difference (WS_2_≈114 ps, WS_2_/Gr≈54 ps). Generally, such a lifetime order of magnitude corresponds to the band‐to‐band recombination process of WS_2_. Therefore, this result indicated that a large part of the photo‐generated carriers in WS_2_/Gr heterostructure is transferred to Gr through interfacial charge transfer. It can be inferred that the photon‐generated carriers in WS_2_ injected into Gr with a near‐unity efficiency.^[^ ^7^ ^]^

As seen in the photograph (Figure 2d) and atomic force microscopy (AFM) image (Figure 2e), the WS_2_/Gr heterostructure displayed clear outlines and edges. According to the AFM height profiles (Figure S4a Supporting Information), the height difference between WS_2_ and Gr was 1.0 nm, while the height difference between graphene and sapphire was 3.7 nm (Figure S4b, Supporting Information). This indicates that the WS_2_ and Gr were couped.^[^ ^10^, ^20^ ^]^ Here, due to the residues and contaminants of the transfer process polymer, and the bubbles and wrinkles generated by large‐area monolayer samples, the height values measured by AFM tended to overestimate the actual thickness.^[^ ^21^ ^]^ Figure 2f shows the Raman spectra of pristine WS_2_ (blue) and pristine Gr (orange), the peaks at 351 cm^−1^ and 417 cm^−1^ were assigned to the in‐plane and out‐of‐plane vibrational modes for WS_2_. The peaks at 1584 cm^−1^ and 2682 cm^−1^ were attributed to the G‐band and 2D band of Gr, respectively. The inset image in Figure 2f shows the Raman mapping at 351 cm^−1^ and 1584 cm^−1^, confirming a good stacking of WS_2_ and Gr. Moreover, the Fermi level of Gr was found to be very sensitive to the position of the G‐band.^[^ ^12^, ^22^ ^]^ Here, the Fermi level of Gr was calculated to be ≈0.1 eV with the position of the G‐band. In addition, power‐dependent Raman experiments were performed on Gr and WS_2_/Gr, respectively. The results are shown in Figure S5 (Supporting Information). In both systems, the G and 2D peaks of Gr were shifted to higher wavenumbers with increasing power, consistent with previous results,^[^ ^23^ ^]^ indicating that both Gr and WS_2_/Gr on sapphire were p‐doped. Referencing the hole doping of a similar structure,^[^ ^24^ ^]^ the Fermi level in this system was determined to be located ≈0.1 eV below the Dirac point of Gr.

**Figure 3:**
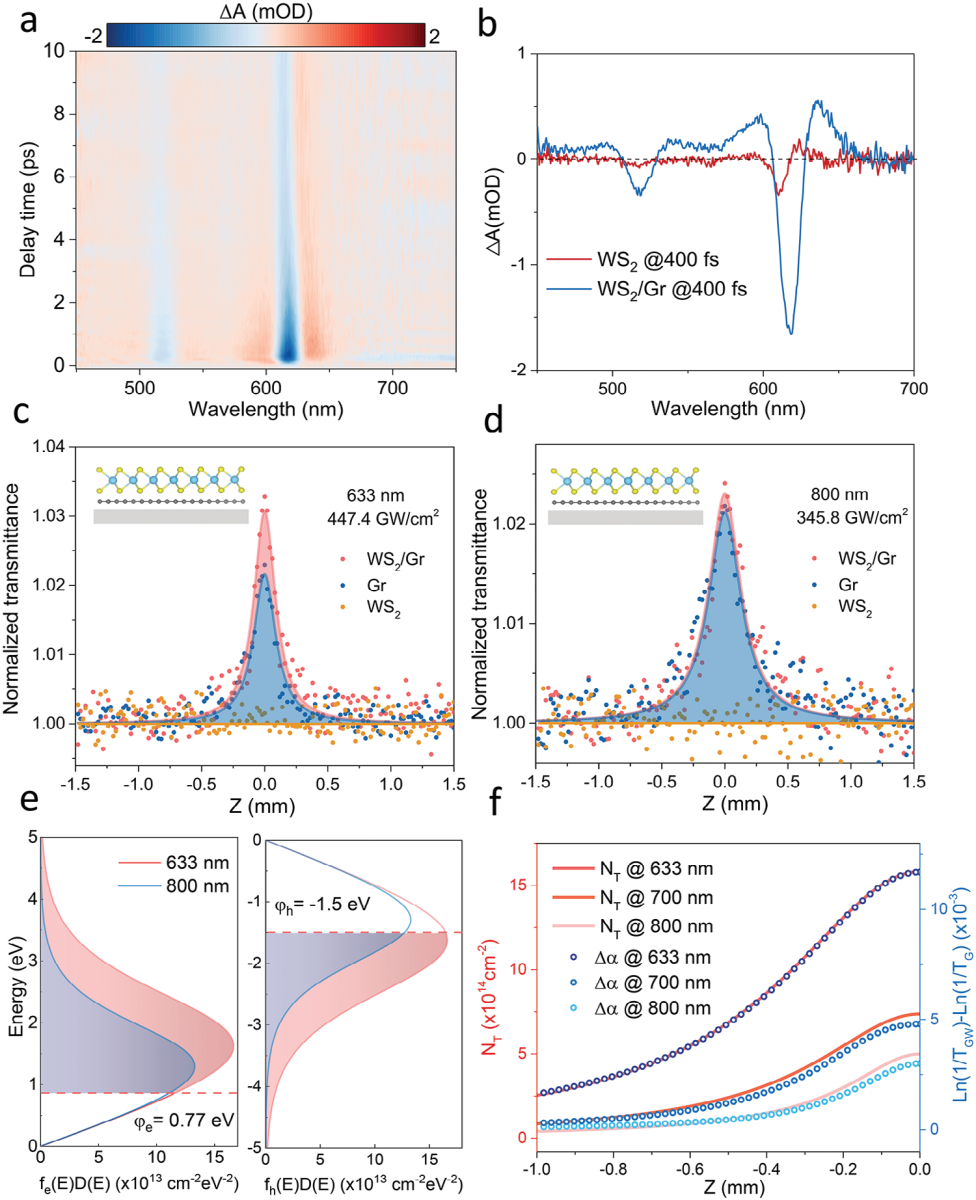
Photo‐thermionic emission (PTE) induced enhancement of saturable absorption of WS_2_/Gr heterostructure by excitation below the WS_2_ bandgap. a) Color plot of TA spectra of WS_2_/Gr heterostructure under 800 nm laser excitation. b) TA spectrum of the WS_2_/Gr heterostructure and WS_2_ at a 400 fs time delay. Open‐aperture (OA) Z‐scan study of WS_2_ monolayers, Gr, and WS_2_/Gr heterostructure with the excitation wavelength of c) 633 nm and d) 800 nm. Inset: the scheme of WS_2_/Gr heterostructure on the transparent sapphire substrate. e) The simulation of FD distribution in 633 nm and 800 nm with the peak optical intensity, where electron and hole barrier were chosen to be 0.77 and −1.5 eV, respectively. f) Calculated the carrier density over the barrier and the experimental absorption coefficient difference between WS_2_/Gr heterostructure and Gr.

TA measurements were performed with the pump photon energy (800 nm) smaller than the A exciton energy. WS_2_ (Figure S6, Supporting Information) and WS_2_/Gr (**Figure** **3**a) showed completely different TA signals at broadband probe wavelengths. Under the same conditions, WS_2_ only showed a weak transient signal at probe@610 nm and @516 nm (Figure 3b), which may be caused by the weak two‐photon absorption of pump light. In contrast, the TA peak position of WS_2_/Gr red‐shifted to 618 nm, and its signal was nearly 6 times larger than that of WS_2_, indicating that the TA signal was caused by the transfer of carriers in Gr to WS_2_ through the PTE effect. Compared with the TA spectrum at 400 nm pump wavelength, the red shift of the A exciton peak of WS_2_/Gr was more obvious, due to the formation of charged excitons in WS_2_. This result implied that the A exciton peak at this time mainly came from charge transfer rather than energy transfer because energy transfer does not form charged excitons.^[^ ^25^ ^]^ Moreover, the WS_2_ characteristic (≈probe 610 nm) kinetic curves of WS_2_/Gr at 400 nm and 800 nm pump wavelengths (Figure S7 Supporting Information), the fitted rise time (188 fs) at 800 nm is larger than that of 400 nm (125 fs). It is further explained that the TA signal with the sub‐bandgap pump comes from the photogenerated carriers transferred from Gr to WS_2_, rather than generated in WS2 because that will result in a similar rise time. The Z‐scan measurements were further performed under sub‐bandgap photon excitation (*hν* < 2 eV, sub‐A‐exciton transition of WS_2_). Figure 3c,d and Figure S9 (Supporting Information) illustrate the NLO absorption response of both the heterostructure and pristine Gr, displaying a symmetrical peak shape indicative of a saturable absorption process. In contrast, the pristine WS_2_ did not exhibit this behavior at the given excitation intensity. Owing to the saturable absorption induced by intense single photon absorption transitions, it is reasonable to suggest that sub‐A‐exciton excitation does not induce SA in WS_2_. Hence, the present observed SA response of WS_2_/Gr was dominated by the Gr layer. Moreover, as anticipated, the SA of WS_2_/Gr can be assuredly enhanced at both excitation wavelengths through a highly efficient PTE process occurring at the interface of WS_2_/Gr. Another intriguing phenomenon is that this enhancement was more pronounced at 633 nm than at 800 nm, which is similar to previous reports.^[^ ^10^ ^]^

A key insight into the contribution of the PTE process to the enhancement of SA was obtained by comparing the number of carriers transferred from graphene to WS_2_ and the difference in absorption coefficients between WS_2_/Gr and pristine Gr. After initial excitation, the carrier‐carrier intraband scattering in graphene leads to the creation of the quasi‐thermalized distribution:^[^ ^10^, ^26^ ^]^

(1) $$f_{e(h)}\left(E\right)=1/\left(\exp\left(\frac{E-\mu_{e(h)}}{k_{B}T_{e}}\right)+1\right)$$

which is illustrated by the separated chemical potentialε_*e*(*h*)_ for electrons and holes at the same temperature *T_e_*. Given that the density of photoexcited carriers (*N_p_*) and the absorbed photon energy (*h*ν) are conserved, ε_*e*(*h*)_ and *T_e_* are as a function of *N_p_* and *h*ν during the ultrafast quasi‐thermal process. According to the conservation of energy and carriers during this process, the hot electron distribution and injection of electrons or holes in Gr can be calculated (Supplementary Section S2) (Figure 3e).^[^ ^10^, ^26^ ^]^ In Figure 3f, N_T_ (the transferred carriers from Gr to WS_2_) was calculated by considering the density of photoexcited carriers at each Z position and extracting the corresponding absorption coefficient difference between WS_2_/Gr and Gr. The excellent agreement between the SA enhancement and N_T_ curve both at 633 nm, 700 nm, and 800 nm revealed that the highly efficient PTE effect directly contributes to improving SA response in WS_2_/Gr through interfacial CT‐induced nonlinear SA enhancement model in Figure 1.

**Figure 4:**
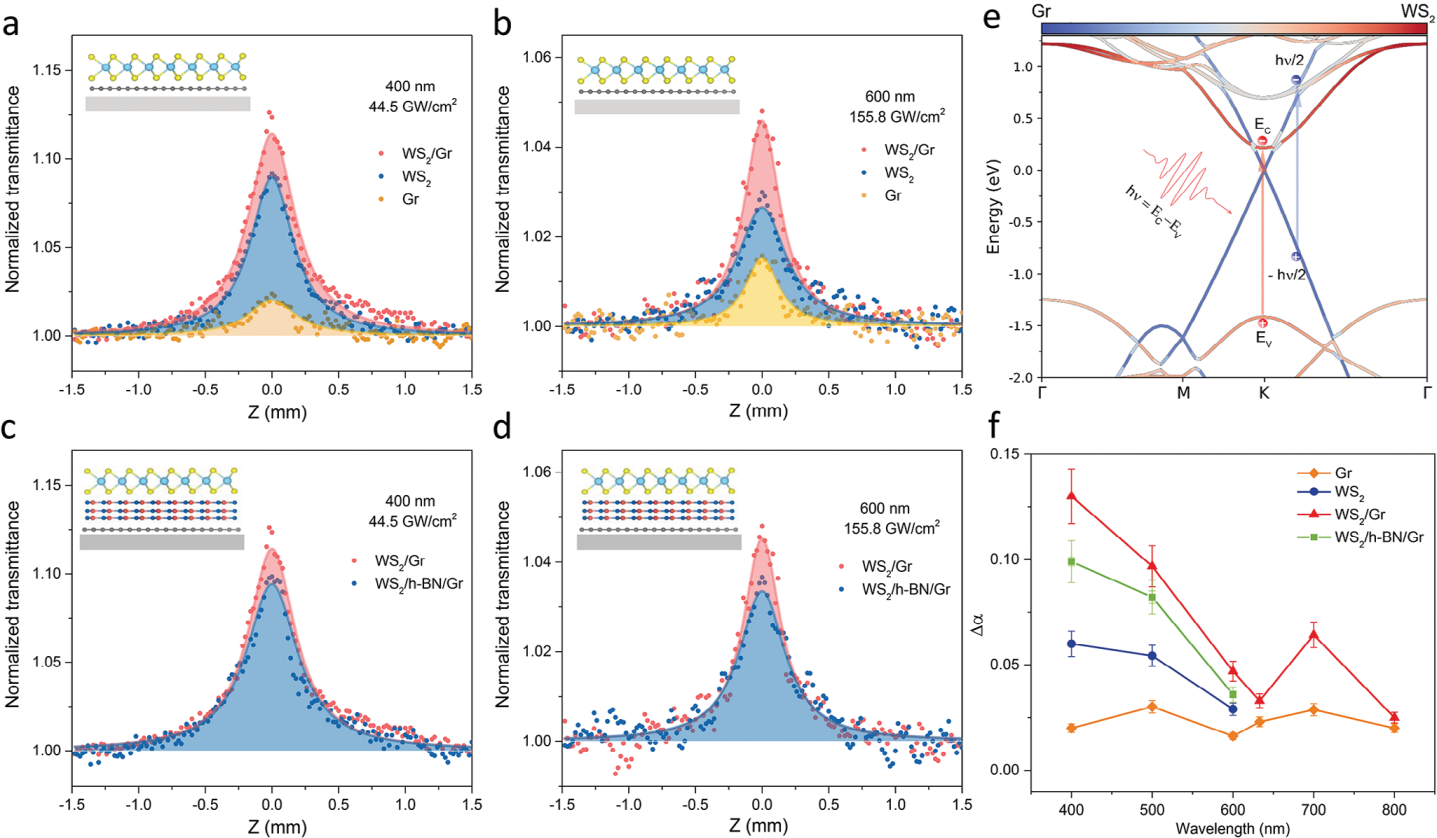
Interfacial charge transfers induced enhancement of saturable absorption of WS_2_/Gr heterostructure under above‐bandgap photon excitation (*hν* > 2 eV, Above‐A‐exciton transition of WS_2_). Open‐aperture (OA) Z‐scan study of WS_2_, Gr, WS_2_/Gr, and WS_2_/h‐BN/Gr heterostructure with the excitation wavelength of a,c) 400 nm and b,d) 600 nm. e) Band structure and schematic diagram of the photoexcitation process in WS_2_/Gr heterostructure. f) Comparison of fitted modulation depth in different wavelengths.

To further evaluate the effect of CT from WS_2_ to Gr on the NLO response of WS_2_/Gr, the Z‐scan was performed with above‐bandgap photon excitation (*hν* > 2 eV, above‐A‐exciton transition of WS_2_) at 600, 500, and 400 nm. As shown in **Figure** **4**a,b and Figure S10 (Supporting Information), WS_2_/Gr heterostructure, pristine WS_2_, and pristine Gr all exhibited saturated absorption. When the excitation photon energy was above the A‐exciton of WS_2_, the NLO response of WS_2_ demonstrated a stronger SA than that of pristine Gr. This can be ascribed to the intense single photon absorption transition near the band edge of WS_2_. Here, WS_2_ exhibited a stronger saturation absorption at 400 nm than 500 and 600 nm, which can be attributed to the stronger linear absorption of WS_2_ at 400 nm.

The effect of interfacial CT on the enhanced NLO response in WS_2_/Gr heterostructure was further analyzed by constructing a WS_2_/h‐BN/Gr sandwich heterostructure. This structure included an additional three layers of h‐BN as an insulating layer. The large band gap of h‐BN (≈ 5.8 eV) acts as a high barrier for carriers in WS_2_ and Gr, preventing direct CT between these two layers.^[^ ^27^ ^]^ Additionally, the excitation photon energies (3.1 and 2.06 eV) are less than the band gap of h‐BN, excluding the SA effect of h‐BN. Only one layer of h‐BN^[^ ^28^ ^]^ can have a strong blocking effect on the band gap state of MoS_2_, so three layers of h‐BN can adequately block the interlayer CT between WS_2_ and Gr. Moreover, the TA experiment (Figure S11a, Supporting Information) of WS_2_/h‐BN/Gr was performed under the excitation of 800 nm. The TA spectrum (Figure S11b, Supporting Information) of WedS_2_/h‐BN/Gr shows a similar intensity of WS_2_, indicating that the h‐BN layer blocked the transfer of most of the carriers from Gr to WS_2_. As illustrated in Figure 4c,d, WS_2_/Gr demonstrated a stronger SA than WS_2_/h‐BN/Gr, emphasizing the key role of carrier transfer for the enhancement of SA in WS_2_/Gr. Compared with the direct CT of WS_2_/Gr heterostructure, the electron transfer caused by the carrier tunneling effect in the WS_2_/h‐BN/Gr system can be neglected, because the carrier tunneling probability is inversely proportional to the band gap size.^[^ ^29^ ^]^ The large band gap of h‐BN obviously does not support significant carrier tunneling. The carriers transferred from WS_2_ to Gr significantly changed its optical properties, thereby enhancing the overall saturated absorption effect of WS_2_/Gr. This was also the reason why the saturation absorption of WS_2_/Gr is stronger than that of WS_2_/h‐BN/Gr. The additional saturable absorption signal of WS_2_/h‐BN/Gr compared with WS_2_ or Gr should come from the linear superposition of WS_2_ and Gr, without the SA enhancement due to carrier transfer.

To identify the possible interlayer coupling CT channel and mechanism, The band structure of WS_2_/Gr was calculated using DFT (Figure 4e). As the photon energy is equal to the band gap of WS_2_, (*hv* = *E_c_* − *E_v_*) the energies of photon‐generated electrons and holes in WS_2_ should be *E_c_* and *E_v_*, respectively, where *E_c_* and *E_v_* are the energies of conduction band minimum and valence band minimum, respectively. The same photon energy can generate the symmetry distribution about the Dirac cone in Gr,^[^ ^4^, ^5^ ^]^ that is, the energy of photon‐generated electrons and holes are half of the photon energy. (hv/hv22 for electrons, −hv/−hv22 for holes) Figure 4e illustrates the photon‐generated carrier energy distribution in WS_2_ and Gr. The electron energy difference between *E_c_* and hv/hv22is > 0.5 eV. (Same as the hole energy difference) This, together with the momentum difference in Brillouin zone, suggests that the transfer of photon‐generated carrier in Gr to WS_2_ is not affected by the state‐filling effects. This is because even considering the carrier thermalization process, most of the transferred carriers from Gr to WS_2_ occupy a state with different energy and momentum from the photon‐generated carriers in WS_2_, and vice versa. According to the previously proposed theory, these transfer processes offer more states to be occupied, leading to the enhancement of saturation absorption.

In order to reflect the proposed model in a quantitative and intuitive manner, the nonlinear optical response parameters of the samples mentioned were extracted by fitting the Z‐scan results. Considering a two‐level system, the SA for OA Z‐scan can be illustrated by the saturable intensity*I_S_* and modulation depth Δα:^[^ ^15^, ^30^ ^]^

(2) $$T=\left(1-\frac{\Delta \alpha }{1+I_{0}/I_{S}\left(1+Z^{2}/Z_{0}^{2}\right)}\right)/\left(1-\Delta \alpha \right)$$

where *I*_0_ is the peak intensity on the axis and *Z*_0_ is the Rayleigh length. As depicted in Figure 4f, WS_2_/Gr demonstrated a higher modulation depth Δα than pristine WS_2_ and pristine Gr, and the modulation depth of WS_2_/h‐BN/Gr was between these two. The stronger linear absorption in the shorter wavelength led to a higher modulation depth, while the saturable intensity *I_S_* demonstrated the reverse tendency (Figure S12a, Supporting Information). As the saturable intensity is defined as half of the required intensity of a completely bleached value,^[^ ^31^ ^]^ a lower saturable intensity means that the heterostructure needs lower optical intensity to reach the saturation state. Gr showed a lower saturation intensity at all wavelengths, followed by WS_2_/Gr, WS_2_/h‐BN/Gr, and WS_2_ in ascending order. Thus, due to the interfacial CT, WS_2_/Gr combines the advantages of Gr and WS_2_ in fast carrier recovery and high modulation depth, respectively, showing its prospects in the field of laser mode‐locking. The traditional NLO propagation equation can be employed to illustrate the SA process:^[^ ^3^, ^17^ ^]^ dI/dIdz′dz′=−(α0+αNLI)I, where *z*′is the propagation distance in the NLO medium, α_0_ and α_*NL*_are the linear and nonlinear optical absorption coefficients, respectively. Figure S12b (Supporting Information) shows the extracted α_*NL*_ as a function of wavelength. The α_*NL*_of pristine WS_2_ reached 10^3^ cm/GW, which is several orders of magnitude larger than the value obtained in the WS_2_ dispersion.^[^ ^32^ ^]^ This is because the calculation of α_*NL*_ was based on the assumption that the NLO medium is a uniform dielectric, and the dispersion form naturally underestimates the α_*NL*_ of each nanosheet. As the thickness of WS_2_/Gr was about twice larger than each composite layer, it is reasonable that the α_*NL*_ of WS_2_/Gr was lower than that of pristine WS_2_ and Gr. The calculated imaginary part of the third order of nonlinear optical susceptibility $Im\chi^{\left(3\right)}$ and the figure of merit (FOM) also showed the same tendency (Figure S12c,d, Supporting Information). However, the heterostructure form is more favorable than the form of multilayer 2D material because the larger modulation depth and shorter ultrafast recovery time of heterostructure have more advantages in the saturable absorber applications.

**Figure 5:**
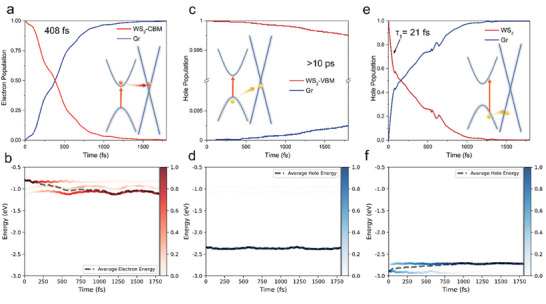
a,c,e) Time‐dependent populations and b,d,f) energy evolutions of electron and hole in WS_2_/Gr heterostructure. (a,b) for electron; c–f) for hole. Schematic diagrams of the corresponding charge carrier transfer process are shown as the inserts in (a,c,e). The black dashed lines in (b,d,e) represent the time‐dependent average energy of the involved electronic states.

Since the enhancement of SA in WS_2_/Gr was essentially dominated by the carrier dynamics, the ab initio NAMD^[^ ^33^ ^]^ was employed to further study the carrier transfer in WS_2_/Gr. Due to the limitations of DFT calculations, only the case of CT from WS_2_ to Gr was considered in this study. **Figure** **5** shows the time‐dependent electron/hole population transfer processes and the corresponding energy evolution processes for the selected states. The electron dynamics at the K point were first studied, as shown in Figure 5a,b, where the initial electron in the CBM of WS_2_ transferred to Gr within 408 fs (fitted with y=e−t/tττ). The hole transfer process at the *K* point was significantly suppressed (Figure 5c,d), i.e., only <1% of the holes in the VBM of WS_2_ were transferred to Gr within the selected time. This indicated that the CT process at the K point is dominated by electron transfer. However, according to the experimental results, the transfer process on a timescale of hundreds of femtoseconds was not sufficient to explain the enhanced saturation absorption process. As the laser pulse width was ≈40 fs, according to the proposed model, a long transfer process would make the short laser pulse insensitive to the extra state of occupation, thereby weakening the enhancement effect of saturation absorption. However, this needs to be confirmed by further pulse‐width‐dependent nonlinear optics experiments. For the hole transfer process at the Γ point (Figure 5e,f), about half of the holes were transferred from WS_2_ to Gr within 20 fs. This is consistent with the previously reported transient angle‐resolved photoelectron spectroscopy results, which suggested that the holes in the WS_2_ valence band would immediately transfer to Gr (within the instrument resolution of 200 fs).^[^ ^24^ ^]^ It is worth noting that this transfer process occurs at an energy position lower than 0.5 eV below the WS_2_ VBM, indicating that the transfer process at the Γ point would be more pronounced when the photon energy is larger than 2.5 eV. This also explains the more efficient SA enhancement is shown at 400 nm (3.1 eV) compared to 600 nm (2.07 eV), further validating our proposed model of carrier transfer enhanced SA.

## 3 Conclusion

The results demonstrate that the considerably enhanced nonlinear optical response can be realized in free‐standing WS_2_/Gr heterostructure with high efficiency under both sub‐bandgap and above‐bandgap photon excitation. Based on the concept of Gr PTE and CT process proposed in previous studies, it was demonstrated that the PTE process occurring at interfacial of WS_2_/Gr heterostructure dominated the saturation absorption enhancement when excited below the WS_2_ A exciton. This result was consistent with the calculations obtained using the proposed model. When excited above the WS_2_ A exciton, the CT from WS_2_ to Gr governed the enhancement process, which was further confirmed by experiments and ab initio NAMD simulations. The results reveal the potential of 2D heterostructures for tailoring the nonlinear optical response of 2D materials and provide insights into the carrier transfer in these systems. In general, the principle of interfacial CT for enhancing nonlinear saturable absorption can be extended and generalized to other 2D heterostructures and other low‐dimensional systems. This may open up new possibilities for exploring novel nonlinear optical phenomena and facilitate applications for ultrashort pulse generation, passive/active tuned photonics devices, and beyond.

## Conflict of Interest

The authors declare no conflict of interest.

## Author Contributions

Y.D.W., Y.W.W., and J.H. designed research. Y.D.W. and Y.W.W. performed the majority of experiments and statistical analysis. C.Y.L. and H.P.L. provided the samples and offered professional advice. L.Z., J.L.K. and W.X.Z. participated in part of the experiments. Y.D.W. drafted the manuscript. Y.W.W., T.Y.X., Y.J.L., X.M.Y., S.X., and J.H. reviewed and edited the manuscript. All authors read and approved the final manuscript.

## Supporting information

Supporting Information

## Data Availability

The data that support the findings of this study are available from the corresponding author upon reasonable request.
