# Supplementary material for: Interfacial Charge Transfer for Enhancing Nonlinear Saturable Absorption in WS2/graphene Heterostructure
Source: Adv Sci (Weinh). 2024 Jan 15;11(12):2306096. doi: 10.1002/advs.202306096 (PMC10966528; doi:10.1002/advs.202306096)
Supplement: Supplementary file 1 — Supporting Information [file ADVS-11-2306096-s001.pdf]

ADVANCED SCIENCE
Open Access

Supporting Information

for *Adv. Sci.*, DOI 10.1002/advs.202306096

Interfacial Charge Transfer for Enhancing Nonlinear Saturable Absorption in WS_2/graphene Heterostructure

Yiduo Wang, Yingwei Wang, Changyong Lan, Li Zhou, Jianlong Kang, Wanxin Zheng, Tianyu Xue, Yejun Li, Xiaoming Yuan, Si Xiao, Heping Li* and Jun He**

Supporting Information

Interfacial charge transfer for enhancing nonlinear saturable absorption in WS_2/graphene heterostructure

Yiduo Wang[1,3], Yingwei Wang [1,3,*] Changyong Lan[2], Li Zhou[1,3], Jianlong Kang[1,3], Wanxin Zheng[1,3], Tianyu Xue[4], Yejun Li[1,3], Xiaoming Yuan[1,3], Si Xiao[1,3], Heping Li[2,*], and Jun He[1,3,*].

1. Hunan Key Laboratory of Nanophotonics and Devices, School of Physics and Electronics, Central South University, Changsha 410083, P.R.China.
2. State Key Laboratory of Electronic Thin Films and Integrated Devices, School of Optoelectronic Science and Engineering, University of Electronic Science and Technology of China, Chengdu 610054, China
3. Hunan Key Laboratory for Super-microstructure and Ultrafast Process, School of Physics and Electronics, Central South University,932 South Lushan Road, Changsha, Hunan 410083, P.R.China
4. Center for High Pressure Science, State Key Lab of Metastable Materials Science and Technology, Yanshan University, Qinhuangdao 066004, China

*Correspondence: YW Wang, E-mail: wyw1988@csu.edu.cn; H Li, oehpli@uestc.edu.cn; J He, E-mail: junhe@csu.edu.cn

This PDF file includes:

Section S1. Methods

Section S2. Calculation of PTE transferred carriers

Section S3. The ab initio NAMD calculations

Figure S1. Schematic diagram of WS_2/Gr in sapphire substrate and transmission spectrum of sapphire.

Figure S2. TA spectra of WS_2 and WS_2/Gr under 400 nm laser excitation

Figure S3. TA spectra of Gr under 400 nm laser excitation

Figure S4. Height profiles of AFM and schematic of the height of the WS_2/Gr heterostructure in sapphire substrate.

Figure S5. Laser power-dependent Raman spectra

Figure S6. TA spectra of WS_2 under 800 nm laser excitation

Figure S7. Kinetic curves of WS_2/Gr at 400nm and 800nm pump wavelengths

Figure S8. Schematic diagram micro-Z-scan system

Figure S9. OA Z-scan with the excitation wavelength of 700 nm

Figure S10. OA Z-scan with the excitation wavelength of 500 nm

Figure S11. TA spectra of WS_2/h-BN/Gr under 800 nm laser excitation

Figure S12. Comparison of fitted NLO parameters

Figure S13. The optimized structure and Brillouin zone of WS_2/Gr heterostructure.

Figure S14. Time evolutions of the energy states in WS_2/Gr heterostructure

Section S1. Methods

WS_2/Graphene Heterostructure Construction

The fabrication of the WS_2/Gr heterostructure used in this study can be found in our previous reports.[1] It is produced by transferring a chemical vapor–deposited (CVD) WS_2 monolayer onto a CVD graphene monolayer which was transferred on a sapphire substrate

Characterization

The optical transmission spectra were obtained with the UV-vis spectrophotometer (UV-2600, Shimadzu). Both the steady photoluminescence (PL) spectroscopy and Raman spectrum was collected by the Renishaw InVia Qontor confocal microsope system. The morphology was investigated by atomic force microscopy (Agilent) techniques. The detail of home-built Pump-probe setup can be found in our previous work.[2, 3]

Micro OA Z-scan

The light source was the Ti:sapphire femtosecond laser system (Spitfire ACE, Spectra-Physics) with a central wavelength of 800 nm, a pulse duration of 40 fs, and a repetition rate of 2000 hz. The detail of home-built open-aperture Z-scan could be found in our previous work.[2] In order to eliminate the influence of dust, wrinkles or uneven areas, based on the original Z-scan, the lens in front of the sample was replaced with a microscope objective (Magnification: 10X; Numerical aperture 0.30), and

the detailed optical setup is shown in Figure S8. With the additional CCD camera and micro-objective (Magnification: 10X; Numerical aperture 0.30), a clean and wrinkle-free area with WS_2/Gr heterostructures could be easily located for Z-scan test. Prior to the sample measurement, we performed the Z-scan test on a clean transparent sapphire substrate to determine the optical intensity that the sapphire do not produce any NLO response. The WS_2 and Gr samples were large-area CVD films uniformly covered on 1x1cm double-polished sapphire substrates. The laser beam waist radius was obtained by fitting the Z-scan curve of the standard sample. The beam waist radius was about 3.5um-5um between 400nm-800nm.

Transient absorption spectra

The femtosecond TA spectra were taken using the Ultrafast System HELIOS TA spectrometer, where the laser source was a Coherent Astrella−1K-F Ultrafast Ti:Sapphire Amplifier (100 fs, 1 kHz, 800 nm) seeded by a Coherent Vitesse oscillator. The pump laser at 400 nm was generated by a BBO crystal.

DFT calculations

All calculations were carried out using the projector-augmented wave method in the framework of the density functional theory, as implemented in the Vienna ab initio Simulation Package (VASP).[4] The generalized gradient approximation (GGA) and Perdew−Burke−Ernzerhof (PBE) exchange functional were used. The plane-wave energy cutoff was set to 500 eV, and the Monkhorst−Pack method[5] with a k-mesh of 6 × 6 × 1 was employed for the Brillouin zone sampling of the WS_2/Gr structure. The convergence criteria of energy and force calculations were set to 10^{-6} eV/atom and 0.001 eV Å−1, respectively. A vacuum region of 15 Å is applied to avoid interactions between the neighboring configurations. The vdW interactions are described with Grimme's DFT-D3 approach.[6] Figure S4a shows the optimized structure of WS_2/Gr, which are constructed by the $2\times2\times1$ supercell of WS_2 matching the $\sqrt{7}\times\sqrt{7}\times1$ supercell of graphene with the lattice mismatch of 1.1%.[7]

Section S2. Calculation of PTE transferred carriers

According to the previous angle-resolved photoemission spectroscopy results,[8] the VBM of WS_2 is 1.5 eV lower than the Dirac point of Gr, corresponding to a hole barrier. And the quasiparticle band gap of WS_2 is 2.27 eV,[9] so the CBM of WS_2 is 0.77 eV higher than the Dirac point, corresponding to an electron barrier. By the Z-scan curves, we can determine the beam waist at different wavelengths. The waist radius at 633 nm is determined to be 4.9 μm, while the waist radius at 800 nm is

determined to be 5.4 μm. Based on the beam waist and the absorbed optical power density, we can determine the injected photon density at each point on the Z axis in the Z-scan. And according to the PTE model in Gr and the barrier height, we can calculate the number of carriers injected into WS_2. As shown in Figure 3d, the calculation results of the PTE model agree well with the effect of saturation absorption enhancement, which shows the connection between carrier transfer and saturation absorption enhancement.

The electrons and holes in graphene that gains enough energy from carrier-carrier scattering could cross the electron barrier ($\varphi_e = 0.77eV$) and hole barrier ($\varphi_h = -1.5eV$), injecting into the conduction band and valence band of WS_2 system, respectively. This part of carriers increased obviously with the excitation wavelength changed from 800 nm to 633 nm. As mentioned above, the modulation depth of the Gr/TMDs is negative correlated with the sum of the distribution function in graphene and TMDs. Hence, the absorption coefficient difference between WS_2/Gr and graphene ($\alpha_d = Ln(1/T_{W/Gr}) - Ln(1/T_{Gr})$) are originated from the transferred carriers ($N_T = \int_{0.77}^{\infty} d\,Ef_e(E)DOS(E) - \int_{-\infty}^{-1.5} d\,Ef_h(E)DOS(E)$) from graphene to WS_2.

Section S3. The ab initio NAMD calculations

The ab initio NAMD calculations are performed using Hefei-NAMD code with phase correction version,[10] which augments the VASP with the NAMD capabilities within time-dependent density functional theory (TDDFT) similar to previous works.[11] To perform different K points Hefei-NAMD calculation,[12] we use the hexagonal unit cell with $3\times3\times1$ k-point grid which including K and Γ highly symmetric points of the WS_2/Gr system. We use velocity rescaling to bring the temperature of the system to 300 K. Then, a 5 ps ab initio molecular dynamics trajectory is then generated from which the time-dependent KS wave functions can be obtained with the time intervals of 1.0 fs. After that, the surface hopping is applied on the basis of fewest switches surface hopping scheme using averaging over 50 different initial configurations and 20000 trajectories for each structure.

Figure S5a shows the optimized structure of WS_2/Gr, which are constructed by the $2\times2\times1$ supercell of WS_2 matching the $\sqrt{7}\times\sqrt{7}\times1$ supercell of graphene with the lattice mismatch of 1.1%. The band structure of heterostructure in Figure 4e demonstrated that the VBM and CBM of WS_2 are on the either side of the Dirac cone of graphene, indicating a type-I band

alignment. That is, the photo-generated electrons and holes in WS_2 will transfer to graphene. According to the schematic diagram of the Brillouin zone in Figure S13b, the K@WS_2 and K@Gr points of the Brillouin zones are folded to the K@HS (K point of the heterojunction), and the Γ@HS (Γ point of the heterojunction) point contains the Γ@WS_2, Γ@Gr, M@WS_2 and M@Gr. This is also evident from the band structure diagram in Figure 4e. We focused on the states near the Fermi level (~ -1 eV) by sampling at the Γ@HS and K@HS, and their energy evolution over time is depicted in Figure S14a and b. Within the given energy range (-3 ~ 0eV), the two red lines at the K point correspond to the CBM and VBM of WS_2, and the two blue lines represent the Dirac cone states of Gr (See Figure S14a). The states evolution at the Γ point differs markedly from that at K point (See Figure S14b). The Gr state appears near -2.7 eV, indicating that the c arrier transfer at the Γ point is dominated by holes and occurs at a deeper energy level.

Table S1. Fitting results of lifetimes

	τ_1 (ps)	τ_2 (ps)	τ_3 (ps)
WS_2	1.28	10.3	114
WS_2/Gr	1.00	8.26	54.0

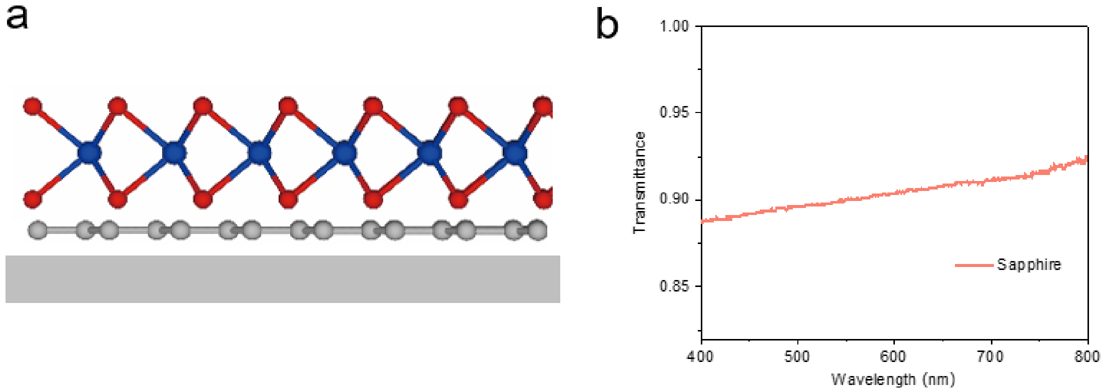

Figure S1. (a) Schematic diagram of WS2/Gr in sapphire substrate. (b) Transmission spectrum of sapphire.

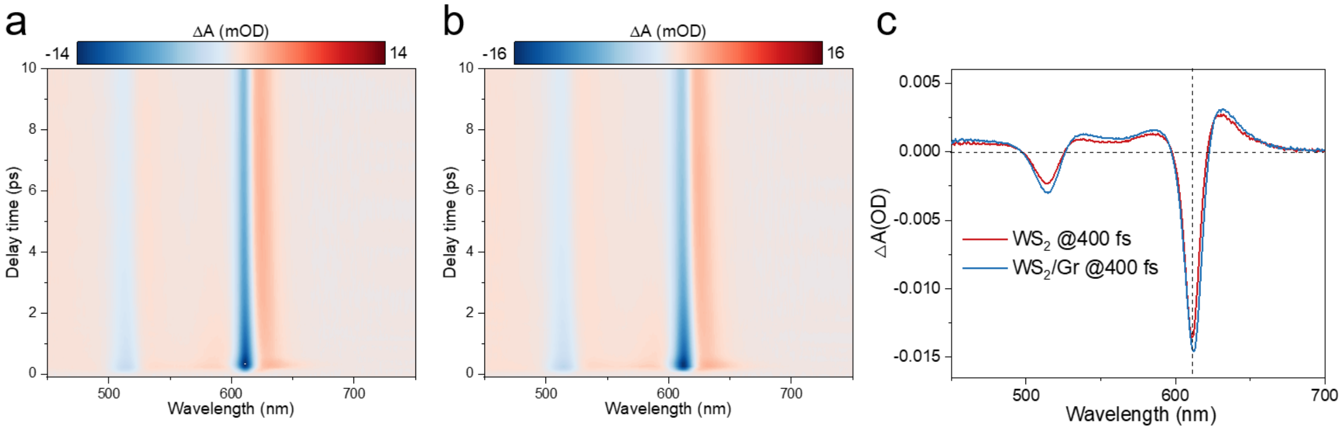

Figure S2. Color plot of TA spectra of (a) WS_2 and (b) WS_2/Gr heterostructure under 400 nm laser excitation. (c) TA spectrum of the WS_2/Gr heterostructure and WS_2 at a 400 fs time delay.

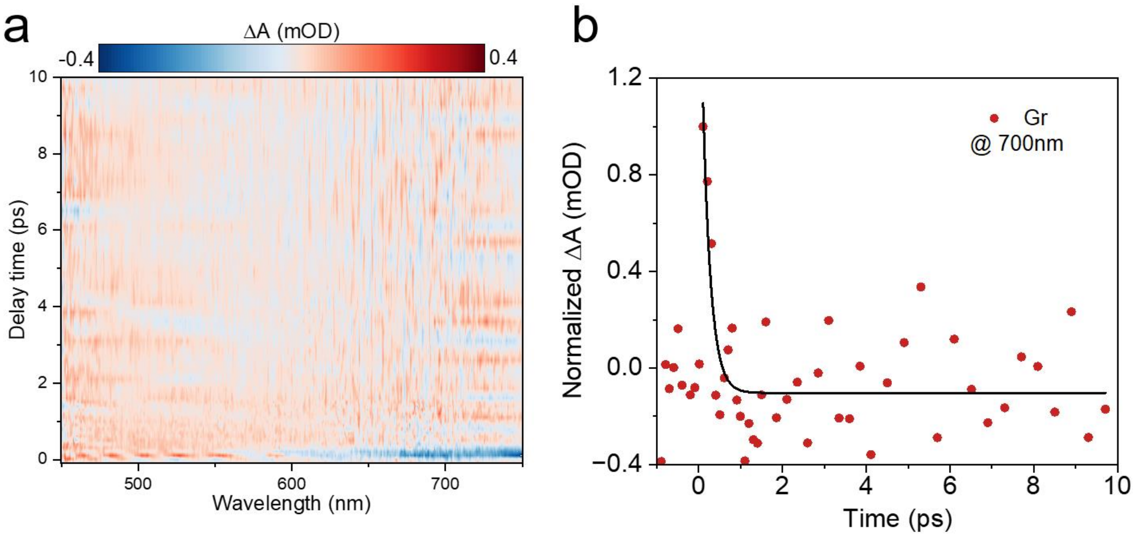

Figure S3. Color plot of TA spectra of (a) Gr under 400 nm laser excitation. (b) Normalized transient dynamics of the Gr probe at 700 nm.

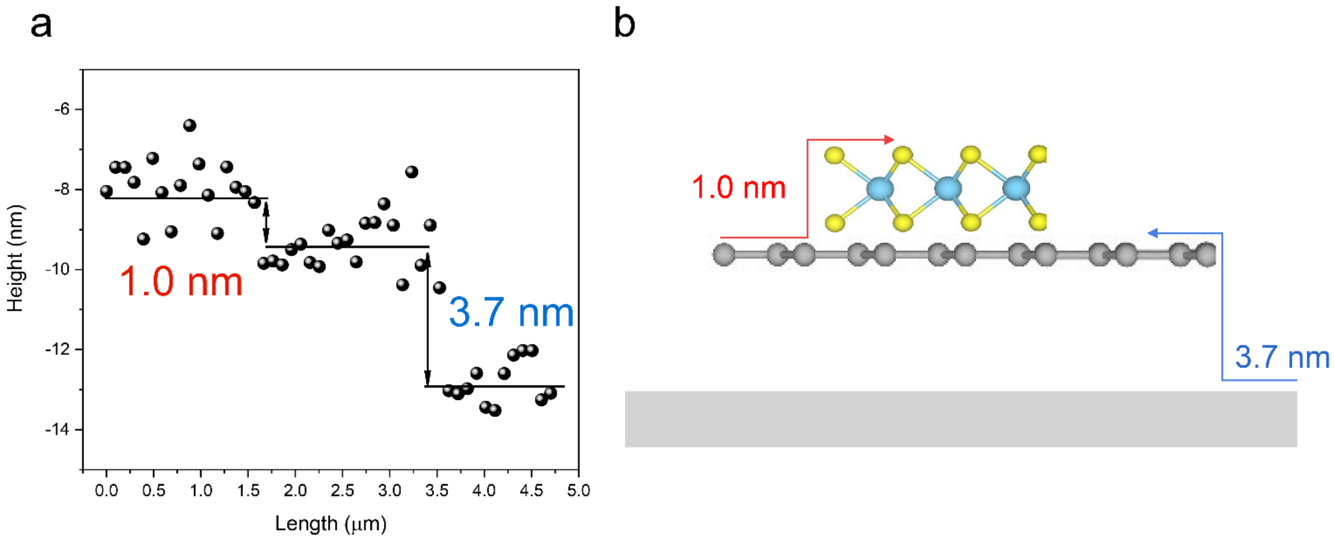

Figure S4. (a) Height profiles, measured by atomic force microscopy, along the dashed white line in Figure 2e. (c) Schematic of the showing the WS_2/Gr heterostructure in sapphire substrate.

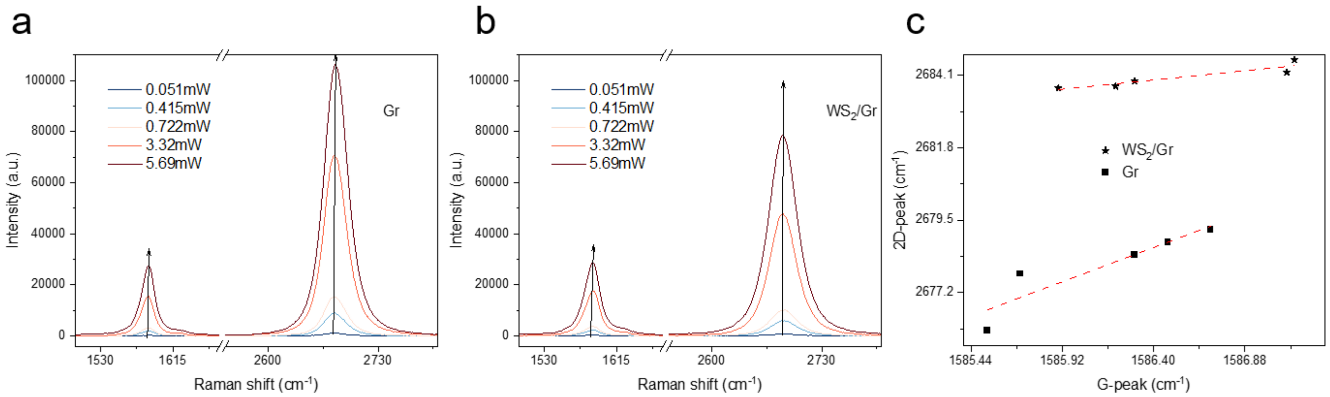

Figure S5. Laser power-dependent Raman spectra of (a) Gr and (b) WS_2/Gr heterostructure. (c) 2D peak position as a function of G-peak position.

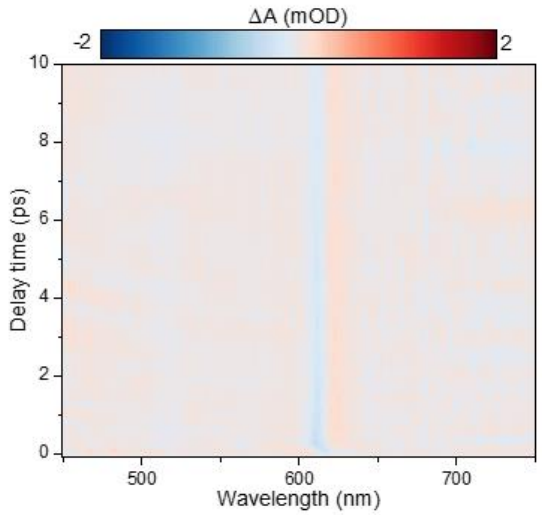

Figure S6. Color plot of TA spectra of WS_2 under 800 nm laser excitation.

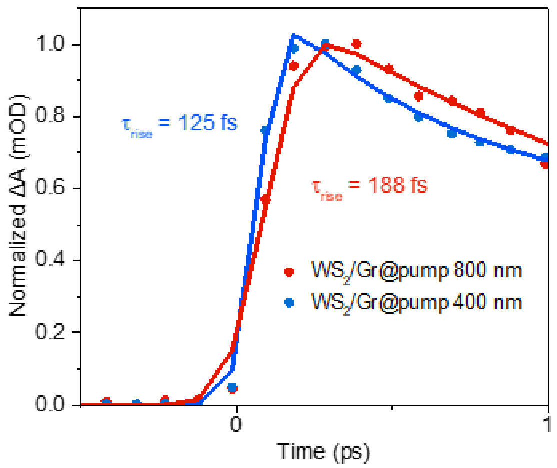

Figure S7. Comparison of WS_2 characteristic (~probe610nm) kinetic curves of WS_2/Gr at 400nm and 800nm pump wavelengths.

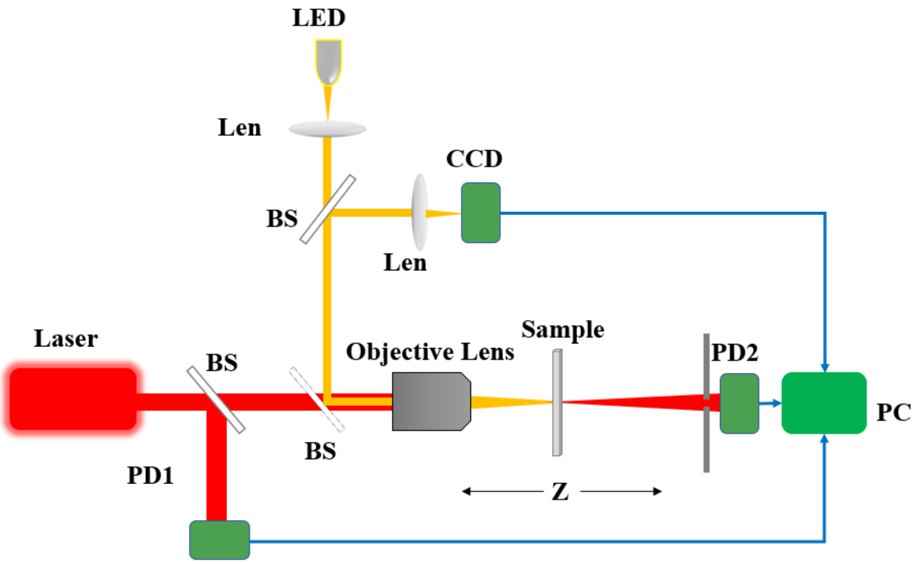

Figure S8. (a) Schematic diagram micro-Z-scan system. PD: photodetector, BS: beam splitter, PC: personal computer.

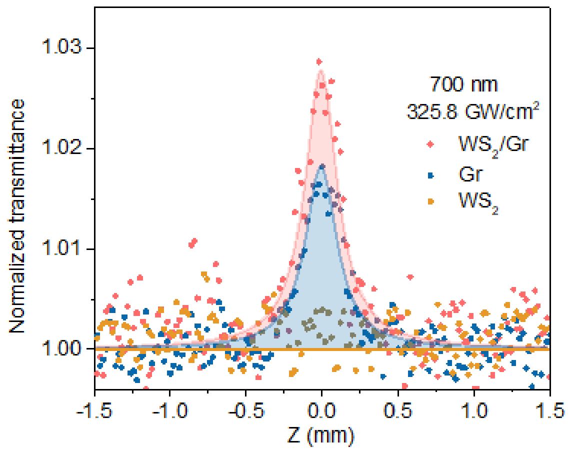

Figure S9. Open-aperture (OA) Z-scan study of WS_2 monolayers, Gr, and WS_2/Gr heterostructure with the excitation wavelength of 700 nm.

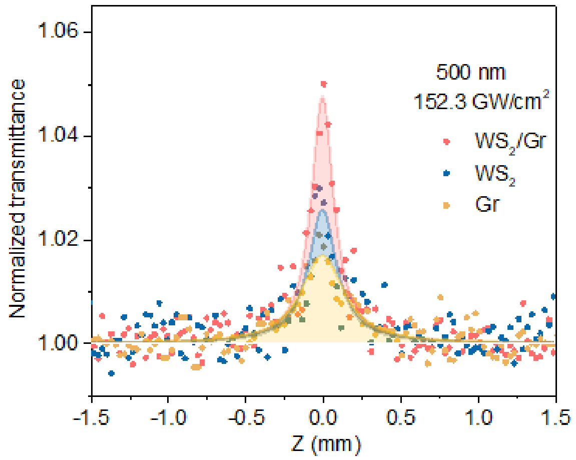

Figure S10. Open-aperture (OA) Z-scan study of WS_2 monolayers, Gr, and WS_2/Gr heterostructure with the excitation wavelength of 500 nm.

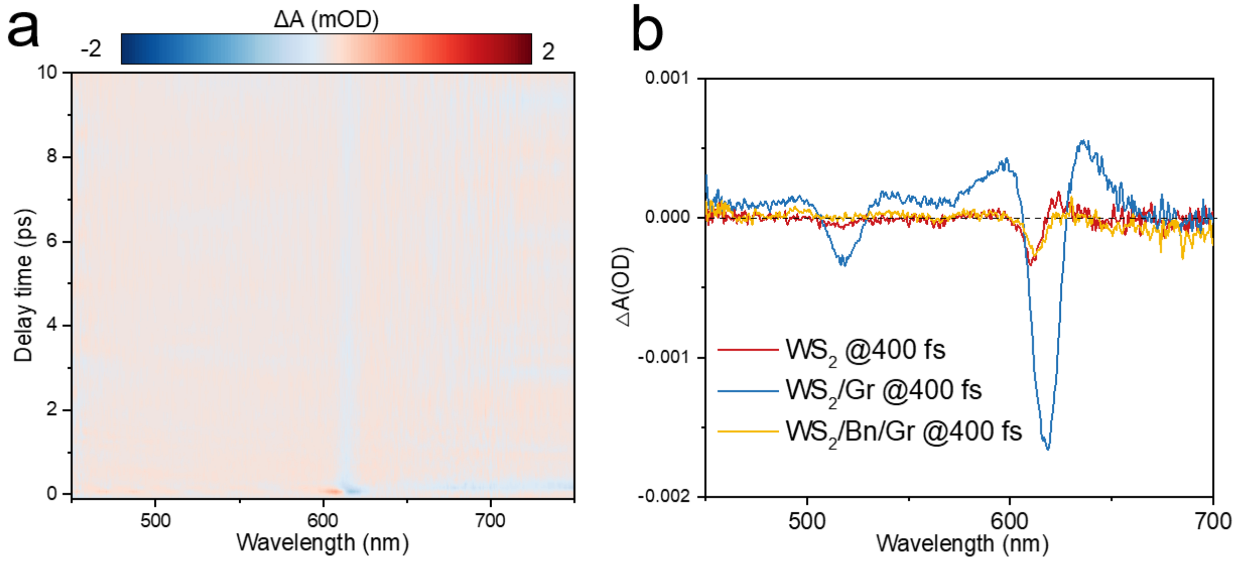

Figure S11. (a) Color plot of TA spectra of WS_2/h-BN/Gr, under 800 nm laser excitation. (b) TA spectrum of the WS_2/h-BN/Gr, WS_2/Gr, and WS_2 at a 400 fs time delay.

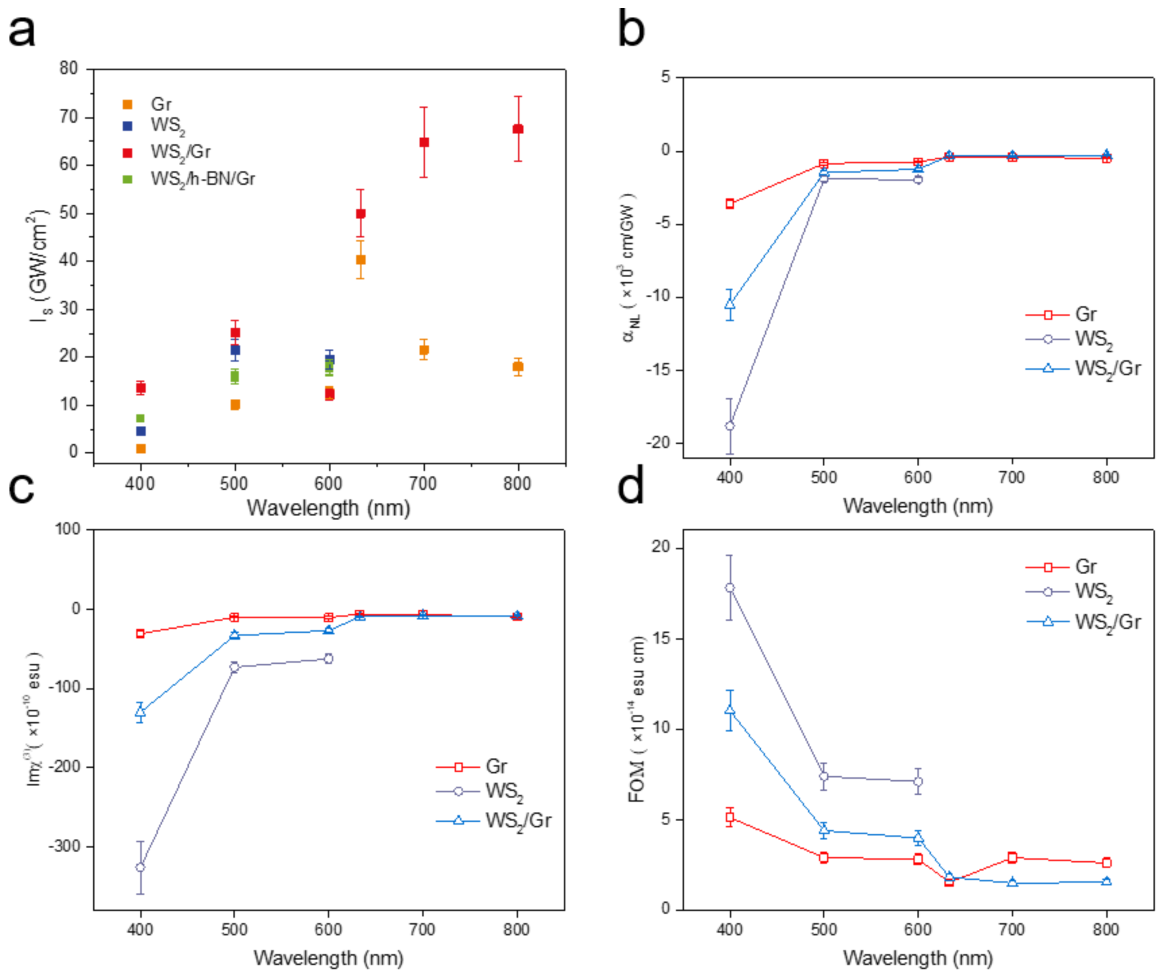

Figure S12. Comparison of fitted (a) saturable intensity, (b) NLO absorption coefficients, (c) imaginary part of the third order of nonlinear optical susceptibility, and (d) figure of merit in different wavelength.

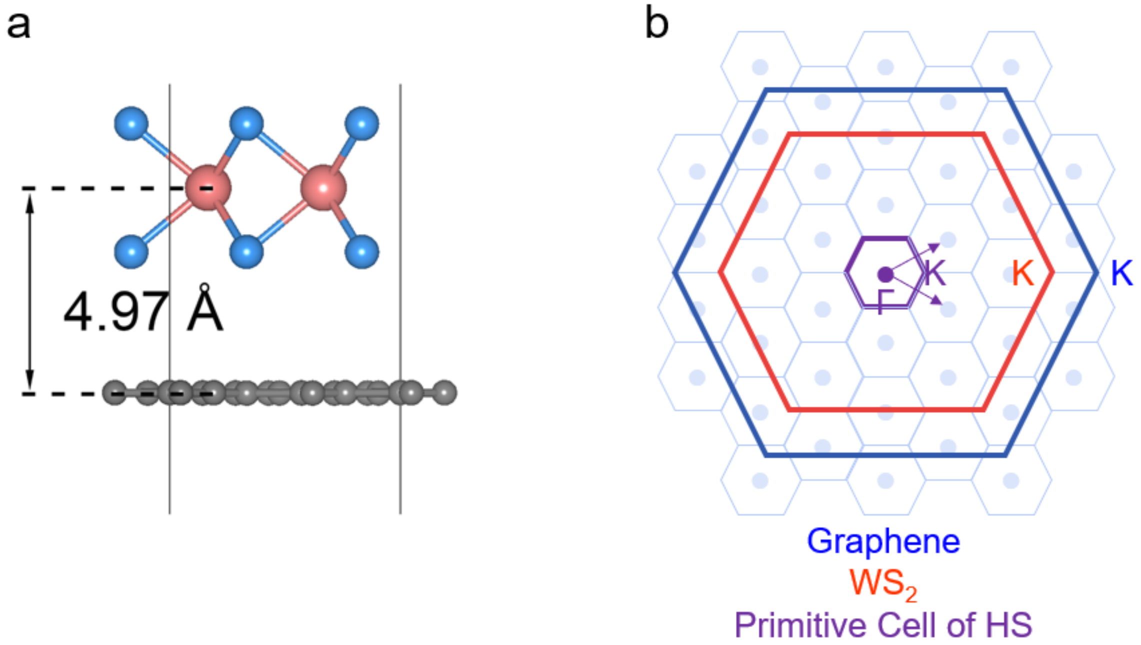

Figure S13. (a) The optimized structure of WS_2/Gr heterostructure. (b) The schematic diagram of the Brillouin zone of WS_2/Gr heterostructure (purple), WS_2 (red), and Gr (blue).

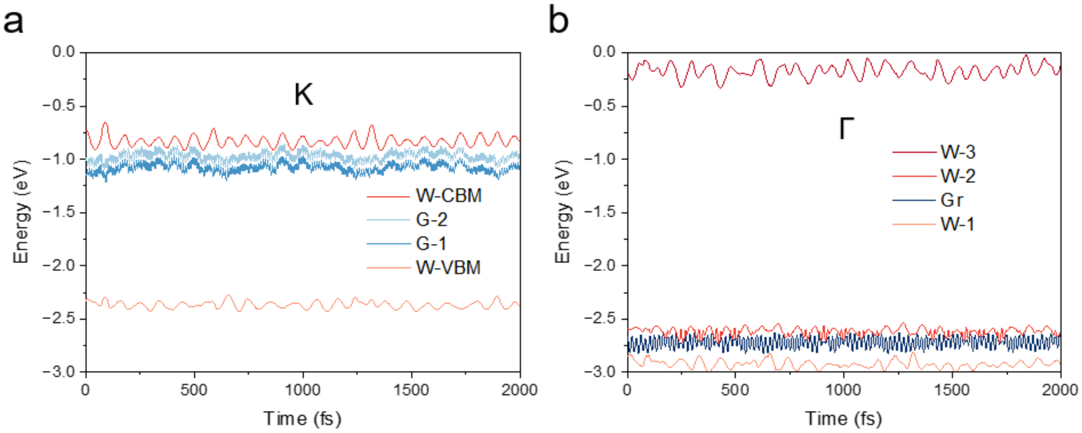

Figure S14. Time evolutions of the energy states in (a) K, and (b) Γ point of high symmetry of WS_2/Gr heterostructure.

References

(1) Du, W.; Li, H.; Lan, C.; Li, C.; Li, J.; Wang, Z.; Liu, Y. Graphene/WS_2 heterostructure saturable absorbers for ultrashort pulse generation in L-band passively mode-locked fiber lasers. *Opt. Express* **2020**, *28* (8), 11514-11523.

(2) Wang, Y.; Wang, Y.; Chen, K.; Qi, K.; Xue, T.; Zhang, H.; He, J.; Xiao, S. Niobium Carbide MXenes with Broad-Band Nonlinear Optical Response and Ultrafast Carrier Dynamics. *ACS Nano* **2020**, *14* (8), 10492-10502.

(3) Wang, Y.; Wang, Y.; Dong, Y.; Zhou, L.; Wei, H.; Long, M.; Xiao, S.; He, J. The nonlinear optical transition bleaching in tellurene. *Nanoscale* **2021**, *13* (37), 15882-15890.

(4) Kohn, W.; Sham, L. J. Self-Consistent Equations Including Exchange and Correlation Effects. *Phys. Rev.* **1965**, *140* (4A), A1133-A1138.

(5) Monkhorst, H. J.; Pack, J. D. Special points for Brillouin-zone integrations. *Phys. Rev. B* **1976**, *13* (12), 5188-5192.

(6) Grimme, S.; Antony, J.; Ehrlich, S.; Krieg, H. A consistent and accurate ab initio parametrization of density functional dispersion correction (DFT-D) for the 94 elements H-Pu. *J. Chem. Phys.* **2010**, *132* (15), 154104.

(7) Liu, X.; Gao, P.; Hu, W.; Yang, J. Photogenerated-Carrier Separation and Transfer in Two-Dimensional Janus Transition Metal Dichalcogenides and Graphene van der Waals Sandwich Heterojunction Photovoltaic Cells. *J Phys Chem Lett* **2020**, *11* (10), 4070-4079.

(8) Aeschlimann, S.; Rossi, A.; Chávez-Cervantes, M.; Krause, R.; Arnoldi, B.; Stadtmüller, B.; Aeschlimann, M.; Forti, S.; Fabbri, F.; Coletti, C. Direct evidence for efficient ultrafast charge separation in epitaxial WS_2/graphene heterostructures. *Sci. Adv.* **2020**, *6* (20), eaay0761.

(9) Raja, A.; Chaves, A.; Yu, J.; Arefe, G.; Hill, H. M.; Rigosi, A. F.; Berkelbach, T. C.; Nagler, P.; Schuller, C.; Korn, T.; et al. Coulomb engineering of the bandgap and excitons in two-dimensional materials. *Nat. Commun.* **2017**, *8*, 15251.

(10) Zheng, Q.; Chu, W.; Zhao, C.; Zhang, L.; Guo, H.; Wang, Y.; Jiang, X.; Zhao, J. Ab initio nonadiabatic molecular dynamics investigations on the excited carriers in condensed matter systems. *WIREs Computational Molecular Science* **2019**, *9* (6).

(11) Yin, Y.; Zhao, X.; Ren, X.; Liu, K.; Zhao, J.; Zhang, L.; Li, S. Thickness Dependent Ultrafast Charge Transfer in BP/MoS2 Heterostructure. *Adv. Funct. Mater.* **2022**, *32* (45), 2206952. Tian, Y.; Zheng, Q.; Zhao, J. Tensile Strain-Controlled Photogenerated Carrier Dynamics at the van der Waals Heterostructure Interface. *J Phys Chem Lett* **2020**, *11* (3), 586-590.

(12) Jiang, X.; Zheng, Q.; Lan, Z.; Saidi, W. A.; Ren, X.; Zhao, J. Real-time GW-BSE investigations on spin-valley exciton dynamics in monolayer transition metal dichalcogenide. *Sci. Adv.* **2021**, *7* (10), eabf3759.
